# Where the Wild Things Are: Transposable Elements as Drivers of Structural and Functional Variations in the Wheat Genome

**DOI:** 10.3389/fpls.2020.585515

**Published:** 2020-09-18

**Authors:** Inbar Bariah, Danielle Keidar-Friedman, Khalil Kashkush

**Affiliations:** Department of Life Sciences, Ben-Gurion University, Beer-Sheva, Israel

**Keywords:** transposable elements, wheat, genome evolution, allopolyploidy, *Triticum aestivum*

## Abstract

Transposable elements (TEs) are major contributors to genome plasticity and thus are likely to have a dramatic impact on genetic diversity and speciation. Recent technological developments facilitated the sequencing and assembly of the wheat genome, opening the gate for whole genome analysis of TEs in wheat, which occupy over 80% of the genome. Questions that have been long unanswered regarding TE dynamics throughout the evolution of wheat, are now being addressed more easily, while new questions are rising. In this review, we discuss recent advances in the field of TE dynamics in wheat and possible future directions.

## Background

Bread wheat (*Triticum aestivum*) is a relatively young allohexaploid species, which has been generated by two subsequent allopolyploidization events that followed the divergence of three diploid wild ancestors: *Triticum urartu* (donor of the A genome), a species from section *Sitopsis*, a relative of today’s *Aegilops speltoides* (donor of the B genome) and *Aegilops tauschii* (donor of the D genome) (Feldman and Levy, 2012; Pont et al., 2019). About 0.5 MYA, a hybridization of the A and the B genome donors that was followed by polyploidization, led to the speciation of the allotetraploid wild emmer, *T. turgidum ssp. dicoccoides* (genome AABB). The initial domestication and cultivation of wild emmer gave rise to the tetraploid lineage, that following selection resulted in the free-threshing durum wheat, *T. turgidum ssp. durum* (genome AABB) (Avni et al., 2017; Pont et al., 2019). The second allopolyploidization event that occurred ~10,000 year ago was the result of hybridization between a tetraploid from the durum wheat lineage and the D genome donor (*Aegilops tauschii)*. This allopolyploidization event resulted in the speciation of bread wheat (*Triticum aestivum*), which today is among the world’s most widely grown crops, providing nearly 20% of daily human caloric intake (Avni et al., 2017; Appels et al., 2018; Pont et al., 2019).

Although wheat is a highly important crop, the challenges in wheat genomics have led to a relatively slow advancement in this field during the beginning of the next generation sequencing (NGS) era as reviewed by Guan et al. (2020). The major obstacle in creating a reference genome draft for bread wheat was the assembly of contigs made from mostly (over 80%) repetitive sequences. The repetitive nature of the wheat genome is mainly the result of a high transposable elements (TEs) content. TEs are DNA fragments capable of increasing their copy number within the host genome mostly through copy and paste (Class I, retrotransposons) or cut and paste (Class II) mechanisms (Wicker et al., 2007). Each class of TEs can be further divided into subclass, orders, super-families, families, and subfamilies, and includes both autonomous and non-autonomous TEs [for more details on the classification system for eukaryotic TEs see Wicker et al. (2007)]. The repetitive nature of the wheat genome, together with its huge size – 17 Gbp, have delayed the generation of the bread wheat genome draft when many other organisms (both animals and plants) have already been sequenced (Bolger et al., 2014; Uauy, 2017; Guan et al., 2020).

## The Era of Wheat Genomics

During the last decade, rapid improvement of DNA sequencing and assembly methods enabled the generation of whole genome assemblies for bread wheat and some of its progenitors. The first genome draft of bread wheat was published by Mayer et al. (Mayer et al., 2014) and was based on chromosome-based sequencing. This genome draft gave the wheat community a first real glance into the complicated wheat genome. In 2017, first genome draft of wild emmer wheat was published (Avni et al., 2017), after a joint effort between industry and academic research groups that has led to the development of an assembly algorithm capable of dealing with highly repetitive DNA sequences. This was a breakthrough in deciphering the large and complex genome of bread wheat and its relatives. Following this publication and the emergence of new sequencing technologies, the sequencing and assembly of all other known wheat relatives became much easier to handle. And indeed, the era of wheat genomics has begun. The sequences of *Aegilops tauschii* (donor of D genome) and *Triticum urartu* (donor of A genome) were published (Luo et al., 2017; Ling et al., 2018), followed by the whole genome draft of bread wheat (Appels et al., 2018) and durum wheat (Maccaferri et al., 2019). The availability of genome drafts for *Triticum* and *Aegilops* species led to a burst of whole genome studies that have assessed the evolution, diversity and structure of the wheat genome, with the association and impact of transposable elements.

## TE Content in Wheat Genomes- Similar, Yet Different?

Transposable elements comprise ~85% of the bread wheat genome, with a relatively even distribution across all 3 sub-genomes (Wicker et al., 2018). Almost 4 million copies belonging to 505 families have been annotated (Appels et al., 2018). The most dominant super-families in the bread wheat genome are *Copia* (Class I), *Gypsy* (Class I), and *CACTA* (class II) (Wicker et al., 2018). Long-terminal repeat–retrotransposons (LTR-RTs) belonging to *Copia*, *Gypsy*, or unclassified super-family comprise 66.6% of the bread wheat genome (Appels et al., 2018). Six families comprise over half of the TE content in bread wheat: *Angela* (*Copia*), *Jorge* (*CACTA*), *Sabrina* (*Gypsy*), *Fatima* (*Gypsy*), *Sumana*/*Sumaya* (*Gypsy*), and *Wham* (*Gypsy*). Although the size of each sub-genome differs (B>A>D), the proportion of TE content remains similar between sub-genomes as well as the composition of super-families and families (Wicker et al., 2018). The difference of the D sub-genome size compared to A and B is mostly due to a lower amount of *Gypsy* elements (Wicker et al., 2018).

Similar TE composition as in bread wheat genome was observed in the rest of the sequenced *Triticum* and *Aegilops* species from different ploidy levels (Avni et al., 2017; Luo et al., 2017; Keidar-Friedman et al., 2018; Ling et al., 2018; Maccaferri et al., 2019). An analysis of TE composition in durum and wild emmer showed the same proportion of TEs in both genomes (82.2%) and highly similar proportions of each TE class/group (e.g., 70% LTR-RTs). About 16% of full-length LTR sequences were found in syntenic positions, meaning that they were not subjected to the rapid turnover of intergenic spaces (Maccaferri et al., 2019). Additionally, similar TE content was observed between the A and B sub-genomes in wild emmer (Avni et al., 2017). In the A and D diploid genome donors, similar TE content was observed (81.4% and 84.4% of the genome in *Triticum urartu* and *Aegilops tauschii*, respectively), with high proportion of LTR-retrotransposons (70.5% and 65.9% in *Triticum urartu* and *Aegilops tauschii*, respectively) (Luo et al., 2017; Ling et al., 2018). While the overall TE content is similar between different *Triticum* and *Aegilops* species from different ploidy levels and between the sub-genomes of allopolyploid wheats (see Guan et al. (2020) for detailed comparison of TE content among wheat species), there is evidence for rapid TE turnover and waves of TE amplification during wheat evolution.

## The Difference Is in the Details

A genome-wide comparative analysis showed that 74% of bread wheat HC (high-confidence) genes are homeologs (conserved between A, B, and D sub-genomes), while most of them are also syntenic between the 3 sub genomes (Appels et al., 2018). Additionally, Wicker et al. (2018) reported that 76% of TE families were found in similar abundance between the A, B and D sub-genomes of bread wheat, meaning less than a twofold change of the proportion between sub-genomes were observed. However, while the gene-based comparison between bread wheat sub genomes indicates high conservation and gene collinearity, the intergenic regions showed almost no sequence conservation between the A, B, and D sub-genomes and almost no conserved TE insertions were identified between them in this study. This phenomena is the result of “TE turnover” that has occurred since the diploid species of the A, B, and D diverged from a common ancestor, meaning these regions have been massively altered by insertions and deletions of TEs (Wicker et al., 2018). Surprisingly, despite the near complete TE turnover, TE family composition between bread wheat sub-genomes remains generally the same with similar proportions between the sub-genomes. However, some TE families showed strong differences in their abundance among bread wheat sub-genomes. Genome wide analysis revealed that most MITE (Miniature inverted-repeat TEs) families were not equally distributed across all three sub genomes of bread wheat. For instance, 70% of *Minos* (*Stowaway* superfamily) insertions were found in the A sub genome, while 79% of *Inbar* (unknown super family) insertions were found in the B sub-genome (Keidar-Friedman et al., 2018). Additionally, strong differences were revealed upon the comparison of TE distribution in the subfamily level. For instance, the highly abundant *Fatima* family of *Gypsy* LTR-RTs has diverged into at least five subfamilies, whereas some subfamilies are found in similar proportions in all bread wheat sub genomes, others have proliferated specifically in the A or B sub-genomes (Wicker et al., 2018).

Upon comparison of 36 MITE families between the bread wheat genome and wheat progenitors (wild emmer wheat and *Aegilops tauschii*), some families presented a copy number similar to the additive value of the parents copy number. However, further analysis has revealed that only 30%–47% of the insertions are common to wild emmer and bread wheat. Keidar-Friedman et al. (2018) suggested that the relatively low proportion of common insertions might be the result of species-specific activity in wild emmer or in bread wheat following hexaploidization and might involve different genomic rearrangements including the deletion of TE containing sequences. The analysis of specific TE insertions demonstrated that while the overall genome content is similar, TEs might still be active and transpose within the host genome. While there is strong evidence for TE activation and TE turnover in wheat, the time frame and the evolutionary events involving the complete TE turnover are still under debate.

## LTR-Retrotransposon Dynamics in Wheat—Amplification Bursts or a Slow Accumulation?

When discussing the time frame for TE activations, the examination of full length LTR- retrotransposons can be used as a clock for insertion age. Due to the transposition mechanism of LTRs-RTs, both LTRs are identical at the time of the insertion into the host genome. LTRs might be diverged due to random mutations and the difference between the LTRs can be used for insertion age estimation (SanMiguel et al., 1998). Using mutations in full length LTR sequences from bread wheat as indicators of insertion age, Wicker et al. (2018) has found the median insertion ages for *Copia*, *Gypsy*, and RLX (unclassified LTR-RTs) to be 0.95, 1.30, and 1.66 million years. Additionally, persistence rate was calculated for full length LTR sequences as the number of elements per 10,000 years that have remained intact until now. The persistence rate analysis is affected by two opposite forces - insertion and deletion and correlates with the full length LTR insertion age distribution. Broad peaks in the persistent rate were revealed for each superfamily, with maxima ranging from 0.6 million years ago for *Copia* in the D sub-genome and up to 1.5 million years ago for RLX. On average, younger full-length LTR insertions from all observed super-families were identified in the D sub-genome, resulting in a shift of ~0.5 million years in the persistence rate distribution relative to the A and B sub-genomes. Additionally, a shoulder in the persistent rate curve was observed for the A and B sub-genomes ~0.5 million years ago, around the time of A and B sub-genomes hybridization.

Commonly, peaks at the age distributions are considered indicators for amplification bursts. Nonetheless, Wicker et al. (2018) suggested that the broad peaks observed for bread wheat represent a slower process of insertion accumulation analogous to mountain range formation, where slow net accumulation over time leads to the creation of large systems, rather than a sudden “burst” of amplification. This claim was supported by the fact that the maximal peak of the age distribution in bread wheat represents a rate of 600 full length LTR copies per 10,000 years. While the mountain range formation explanation indeed fits the results, we suggest that amplification bursts cannot be ruled out as a possible explanation for the observed TE dynamics. Although 600 new copies per 10,000 years is a relatively slow increase, it can’t be deduced that the amplification rate was constant along this time frame, and short sharp bursts can be masked by this specific analysis where the persistence rate is calculated as number of copies per 10,000 years. Additionally, the persistence rate is affected by both rate of insertion and rate of deletion. Several studies performed on newly synthesized wheat allopolyploids indicate possible activation of transposable elements (TEs), together with reproducible elimination of TE-containing sequences in the first generations of the new polyploid species (Shaked et al., 2001; Kraitshtein et al., 2010; Yaakov et al., 2013). Amplification burst accompanied by massive sequence elimination might result in low net increase in new copies, considering that newer TE insertions are considered more susceptible to removal by homology‐dependent illegitimate recombination (Schrader and Schmitz, 2019). Comparative genomics between various wheat species and accessions together with studies focusing on synthetic wheat polyploids might shed light on the timing and the mechanisms involve in LTR- retrotransposons activations in wheat.

Dating LTR-retrotransposon insertions in *Triticum urartu* genome revealed an amplification wave of the *Gypsy* superfamily over more than ~1 million years ago and of the *Copia* super-family less than ~1 million years ago (both after the divergence of A and B genomes). Sequence alignment of *T. urartu* chromosome 7 vs bread wheat chromosome 7A showed an alignment of 91% of *T. urartu* to bread wheat. The remaining unaligned regions were LTR sequences. This analysis showed that the A genome has gone through large structural rearrangements that involved TEs both before and after the polyploidization event of bread wheat speciation. A comparison of the *T. urartu* chromosome 3 vs bread wheat chromosome 3B showed both experienced an LTR retrotransposon amplification wave ~1 million years ago, while bread wheat 3B had even a larger wave of amplification 0.1 million years ago (Ling et al., 2018).

Based on the age distribution of full length LTR-retrotransposons in wild emmer wheat, Avni et al. (2017) suggested a wave of *Gypsy* and RLX elements amplification dated to ~1.5 million years ago (similar patterns in both A and B sub-genomes), a wave of *Copia* LTR amplification dated to ~0.5 million years ago, around the time of tetraploidization and another wave of *Copia* amplification around 1.2 million years ago in B sub-genome of wild emmer wheat. While the comparison between wild emmer and durum revealed high similarity in TE content and synteny of full length LTRs, no further examination of TE turnover was done in this case (Maccaferri et al., 2019). Thus, it is possible that the relatively short time separating wild emmer and durum led to high similarity of the intergenic space. However, these results might also indicate the same pattern seen in bread wheat sub-genomes, where the total number of TEs remains quite the same despite their possible activity.

In the *Ae. tauschii* genome, dating of LTR-retrotransposon insertions revealed an amplification wave ~1 million years ago. When zooming in, LTR families have gone through amplification and silencing over the past 3 million years. LTR families in the proximal regions were older than those in the distal regions, probably due to faster removal of DNA in distal regions (Luo et al., 2017).

## TE Content Depends on the Chromosomal Context

When examining the density of TEs across chromosome arms in bread wheat, the proportion of TE was lower in distal regions (~73%) compared to proximal and interstitial (~89%). The different TE families showed variation in their distribution across chromosomes in bread wheat. For example, *Angela* (*Copia*) and *Caspar* (*CACTA*) families are enriched in telomere regions while *Sabrina* (*Gypsy*) and *Jorge* (*CACTA*) are enriched in central parts of chromosomes arms (Wicker et al., 2018). For *Triticum urartu* and *Aegilops tauschii*, the distribution of specific TE super-families across the chromosomes was described, and similar to what was observed for bread wheat, *Copia* elements were enriched in both telomeric and sub-telomeric regions, while *Gypsy* elements were enriched in pericentromeric and centromeric regions (Luo et al., 2017; Ling et al., 2018). The centromeres in bread wheat and in *Aegilops tauschii* are characterized by unique TE content and *Cereba* (*Gypsy*) elements were found to be concentrated in centromeric regions in both species (Luo et al., 2017; Wicker et al., 2018).

Both Mayer et al. (2014) & Wicker et al. (2018) showed that LTR-retrotransposons and *CACTA* (Class II) elements dominate the intergenic regions in bread wheat, while Non-LTR elements and MITEs, mostly *Tourist* and *Stowaway* super-families, are highly abundant in genic regions. However, there are exceptions to the rule, as groups or super-families cannot always predict TE enrichment in the vicinity of genes. Keidar-Friedman et al. (2018) has found that while ~52% of all MITE insertions retrieved from four different wheat genomes (bread wheat, wild emmer, *Triticum urartu* and *Aegilops tauschii*) are located within or in close proximity to protein-coding genes, some MITE families showed different distribution patterns, i.e., ~90% of *Inbar* (unknown superfamily) insertions were found in retrotransposon sequences (Keidar-Friedman et al., 2018).

## Impact of TEs on Gene Expression and Function

Due to the high abundance of TEs in the wheat genome, it is not surprising that almost all bread wheat genes are flanked by TEs in their direct vicinity. The unique pattern of TE distribution observed in genes vicinity has led Wicker et al. (2018) to examine the possible role of TEs in gene regulation. An enrichment analysis for TEs in genes promotors on a database of expression modules sharing a common expression profile across exhaustive set of wheat RNA-seq data did not reveal strong association between specific TE family found in genes promotors and expression module. Additionally, a study by Ramírez-González et al. (2018) examined the impact of transposable elements in gene promoters on differential expression patterns of homeologs triads (homeologs with a 1:1:1 correspondence across the three sub-genomes) in bread wheat and found no correlation between TEs presence in genes promotors and altered expression patterns of the triads in leaves. However, more dynamically expressed triads across different tissues showed higher TE abundance in the vicinity of the translation start site. Those observations led Ramírez-González et al. (2018) to suggest that the promoter TE landscape might affect gene expression in a tissue specific manner as cis-regulatory elements or through other epigenetic mechanisms, rather than having a general effect on the differential gene expression across all tissues (Ramírez-González et al., 2018).

Although on a genome wide level no association was found between TE families in the direct vicinity of genes and stress response (Wicker et al., 2018), there is evidence for TE mediated gene regulation networks during stress response in wheat. Poretti et al. (Poretti et al., 2020) showed that MITEs from the *Mariner* superfamily contribute to the large diversity of miRNAs during the wheat immune response to the powdery mildew pathogen. These findings together with the fact that MITE sequences are prone to domestication into miRNA and are frequently associated with genes, led Poretti et al. (2020) to suggest that MITE domestication into miRNA precursors might have an important role in driving miRNA functional innovation in wheat.

TE insertions can be found within genes and thus might have an effect on gene regulation both in the level of transcription and post-transcriptionally (Schrader and Schmitz, 2019). Several studies have reported on an association between a specific TE insertion into a gene and the levels of gene expression in wheat. In one case, the insertion of a *Tourist* MITE into the 3’ UTR of a heat shock protein gene (TaHSP16.9-3A) has led to up-regulation of the gene expression following heat treatment (Li et al., 2014). In other cases, the presence of a TE insertion within the exon 6 of a gene that encodes for 5-formyltetrahydrofolate (Domb et al., 2019) and an insertion into the 5’ UTR of a gene coding for chalcone synthase (Xi et al., 2016) were correlated with lower transcript levels relative to accessions that did not contain the insertions. However, the complex regulation of these genes did not allow validation or ruling out of the possible effect of TEs insertion on transcript level. These cases suggest that the regulation of genes in wheat is quite complicated and the association of transposable elements is not always clear. Additional studies are needed to elucidate the possible role of TEs in the regulation of wheat genes.

TEs might directly impact gene function by insertion into protein-coding sequences, which usually lead to mutations and generation of modified proteins (Rebollo et al., 2012; Zhao et al., 2016; Jiang et al., 2019; Schrader and Schmitz, 2019), while intronic insertions can create new alternative splice variants or go through exonization and/or intron retention events (Lev-Maor et al., 2003; Krull et al., 2005; Schmitz and Brosius, 2011; Dubin et al., 2018; Keidar et al., 2018). Hundreds of MITE insertions were identified in the transcriptome of bread wheat and wild emmer. While most MITE insertions were located in the UTRs of the transcripts, ~13% of the insertions in bread wheat and ~19% in wild emmer are found, at least partly, within coding regions (Keidar-Friedman et al., 2018). The MITE-containing transcripts are usually longer relative to other transcripts of the same gene that do not contain the MITE insertion due to alternative splicing, while the predicted protein length can be shorter, longer, or the same between the different transcripts (Keidar-Friedman et al., 2018). Like MITEs, insertions of *Au* SINEs, a non-LTR retrotransposon family highly abundant in many plant species, are found in hundreds of bread wheat transcripts. *Au* SINE containing transcripts of protein-coding genes were found to be shorter when compared to transcripts that do not include the *Au* SINE insertion, and if translated will lead to shorter proteins (Keidar et al., 2018). In several cases, the insertions of *Au* SINEs into introns led to intron retention or to exonization, which led to the generation of alternative transcripts (Keidar et al., 2018). Although these alternative transcripts were expressed in a much lower level than the regular transcripts, they might lead to creation of new modified proteins or act as regulators of these genes.

Although TE insertions are known to be prevalent within and in vicinity to wheat genes, currently, there are only few known examples for direct effect of TE insertions on phenotype in wheat. Jiang et al. (2019) identified a new allele to the Q gene in Tibetan semi-wild wheat (Q^t^) with a transposon insertion in exon 5. The Q^t^ allele most likely originated from the domesticated Q allele and was found to be unique to Tibetan semi-wild wheat, a potential de-domesticated common wheat subspecies. The Q gene influences many domestication related traits in wheat, such as rachis fragility (Simons et al., 2006). While the TE insertion did not have an effect on the Q gene expression level, it led to abnormal function of the transcribed protein, resulting in brittle rachises, thus contributing to re-acquisition of wild traits in this sub-species (Jiang et al., 2019). Additional example is the *VRN1* homologs which show high allelic variation in wheat. Various mutations in *VRN1*, including mutations induced by TE insertions, were found to influence heading stage in wheat, as reviewed by Shi et al. (2019).

## The Possible Role of TEs in Wheat Genomic Rearrangements

In addition to allelic variation that can be created due to TE insertions, TE activity might also result in genomic rearrangements due to double strand breaks triggered upon insertion or excision or due to alternative transposition events (Gray, 2000; Hedges and Deininger, 2007; Krasileva, 2019). Furthermore, the highly repetitive nature of TEs can lead to disruptive interactions during both meiotic recombination and DNA repair processes, resulting in a variety of genomic rearrangements (Gray, 2000; Devos et al., 2002; Ma, 2004; Hedges and Deininger, 2007; Krasileva, 2019). Due to the absence of high-quality wheat genome assemblies, previous comparative analyses between different wheat species and cultivars have focused on sequence analysis of structural variants of a few bp up to several Kb (Liu et al., 2016; Montenegro et al., 2017). So far, the assessment and identification of large scale chromosomal rearrangements in wheat relied mainly on C-banding (Friebe and Gill, 1994; Badaeva et al., 2007), SNP (single nucleotide polymorphism) analysis (Cavanagh et al., 2013; Hao et al., 2017) and FISH (Schneider et al., 2003; Du et al., 2017; Huang et al., 2018). While FISH provides a simple and efficient way to screen large numbers of wheat accessions in order to identify large scale rearrangements, it is limited in regard to the characterization of the rearrangements break points.

Recent technological developments in sequencing and assembly methods provide a basis for the generation of high-quality *de novo* assemblies of complex plant genomes (Uauy 2017). Application of the new technologies to different accessions of diploid and allopolyploid wheat might be the first step toward revealing the molecular mechanisms for large scale rearrangements in the wheat group. Recent studies relied on high-quality sequence assembly of several wheat species and cultivars for the conduction of large-scale comparative sequence analysis, which enabled the identification of large structural variations (Dvorak et al., 2018; Huo et al., 2018; Thind et al., 2018; Bariah et al., 2020; Keidar-Friedman et al., 2020). Unequal crossing over and double-strand break repair *via* Non-Homologous End-Joining (NHEJ) was suggested as a possible mechanism for large scale indels (insertions-deletions) between different wheat species and cultivars (Thind et al., 2018; Bariah et al., 2020). While there is evidence for the possible involvement of TEs in wheat genomic rearrangements, the extent and underlying mechanisms of genomic rearrangements in wheat remain largely unknown (Bariah et al., 2020).

## TEs and Populations Genetics Diversity

Crop domestication involved recurrent selection to increase the frequency of desirable traits, leading after thousands of years to dramatic loss in genetic variation (Bevan et al., 2017). Wild relatives of domesticated wheat are recognized as a great potential source for crop improvement in face of growing global population, environmental changes and increasingly challenging growing conditions (Bevan et al., 2017; Haas et al., 2019). Due to their activity and involvement in genomic rearrangements, TEs are important source of intra-specific genetic variation (Dubin et al., 2018). Although many studies have focused on phenotypic and genetic variations in natural populations of wheat wild relatives, only few recent studies have focused on TE dynamics in natural wheat populations.

Different TE based genetic markers were implemented in order to examine the genetic diversity of wild emmer populations from Israel and Turkey and the phylogenetic relationship between populations. According to studies using retrotransposon-based marker methods (IRAP and REMAP) (Vuorinen et al., 2018), MITE-based markers (TD) (Domb et al., 2017) and additional TE based genetic markers (Venetsky et al., 2015; Domb et al., 2019), the genetic distances between wild emmer populations did not correlate with their geographical distances. While most of the populations clustered separately, in several studies some of the populations did not separate clearly, which indicates high levels of polymorphism within the analyzed populations (Vuorinen et al., 2018). An additional study examined the genetic diversity in Turkish wild and domesticated emmer using iPBS-retrotransposon markers and did showed clear clustering of tetraploid wheat wild and domesticated accessions based on their geographic origin and species (Arystanbekkyzy et al., 2019).

Several studies have focused on TE diversity in wild emmer populations in Israel. TEs from different classes and families were shown to vary in their copy number within and between 5 Israeli populations. The *Eos* (*Stowaway*) family for example, showed almost 3 times the amount of elements in Mt. Hermon and Tabja populations compared to Amiad populations, while *Balduin* (*CACTA*) had a higher copy number in Amiad compared to Mt. Hermon. It has been suggested that MITEs retained their activity in wild emmer due to the high polymorphism levels found in these populations (an average of 78.8%) (Domb et al., 2017). TEs have been shown to generate allelic variation in genes of Israeli populations of wild emmer (Domb et al., 2017; Domb et al., 2019) as some alleles contain insertions of TEs within their introns, exons, or near genes. Additionally, TEs are massively targeted by CpG methylation and were found to be involved in population-unique methylation patterns between the 5 different Israeli wheat populations (Venetsky et al., 2015).

## Concluding Remarks

The role of TEs as regulators of genes in plant genomes has been well established (Slotkin and Martienssen, 2007; Bourque et al., 2018; Dubin et al., 2018) and there is evidence for their involvement in chromosomal rearrangements (Bennetzen, 2005; Parisod et al., 2009; Fedoroff, 2012; Sigman and Slotkin, 2015; Kent et al., 2017) and generation of genetic diversity (Oliver et al., 2013; Dubin et al., 2018; Venkatesh, 2020). As the major component of the wheat genome, it is now clear that TEs take place in different structural and functional variations of the genome. However, there is still a lot to learn about TE dynamics in wheat genomes and the involving mechanisms.

## Future Perspectives

The characterization of TE distributions in wheat genomes has revealed family and subfamily unique insertion patterns, including sub-genome specificity and preferential insertion within specific genomic contexts (Keidar-Friedman et al., 2018; Wicker et al., 2018). The similarity in TE content and context between the bread wheat sub-genomes and between wheats from different species is surprising considering the near complete TE turnover occurred since the three diploids genome donors diverged from a common ancestor (Avni et al., 2017; Luo et al., 2017; Keidar-Friedman et al., 2018; Ling et al., 2018; Maccaferri et al., 2019). We suggest that integration site selection together with epigenetic mechanisms leading to equilibrium in TEs copy numbers might explain these interesting phenomena. New insights regarding the underlying mechanisms for TE equilibrium in genomes might arrive from the implementation of mathematical and physical models on experimental data (Roessler et al., 2018; Bourgeois and Boissinot, 2019; Bousios et al., 2020).

The wheat group offers an ideal system to study the evolution of polyploidy due to the ability to conduct comparative analyses between available species with different ploidy levels and since newly formed wheat allopolyploids can be easily produced in the greenhouse (Li et al., 2018). In light of the recent developments in wheat genomics, the examination of new models based on whole genome sequencing, epigenetic, and 3D analysis (Concia et al., 2020) is now technically possible for newly synthesized allopolyploids. Studies of TE dynamics in newly synthesized wheat allopolyploids might deepen our understanding of the effect of perturbation on host: TE dynamics (Roessler et al., 2018) and on the possible effect of the 3D genome organization on TEs insertion sites and propagation across the nucleus (Bousios et al., 2020).

The ability to conduct comparative analysis on the first generations of newly synthesized allopolyploids and among a variety of wheat accessions will also provide the wheat TE community with new tools to address long-awaited questions regarding TE dynamics following polyploidization events and during wheat evolution, as well as their possible role in the adaptation of these allopolyploids. This includes the timing and possible triggers for TE activation, the possible role of TEs in genomic rearrangements and the effects TEs might have on the 3D genome organization of wheat genomes.

Gaining a better understanding of TEs impact on genetic diversity and speciation in wheat is also important in the context of wheat improvement. Alongside their potential use as genetic markers for wheat molecular breading (Venkatesh, 2020), TEs insertions might have an effect on agricultural important traits (Domb et al., 2019; Jiang et al., 2019; Poretti et al., 2020). We believe that additional genome sequencing projects of various cultivated and wild wheat accessions, like the 10+ Wheat Genomes Project (http://www.10wheatgenomes.com/), together with gene expression analysis will contribute a lot to the efforts to shed light on the possible impact of TEs on gene expression and function in wheat and possibly to wheat improvement.

## Author Contributions

IB wrote the review. DK-F wrote the review. KK wrote, corrected, and finalized the review for submission.

## Funding

This work was funded by Israel Science Foundation (grant # 322/15) to KK. The funder has no any role on data generation or discussion.

## Conflict of Interest

The authors declare that the research was conducted in the absence of any commercial or financial relationships that could be construed as a potential conflict of interest.
